# Joint-preservation surgery for bone sarcoma in adolescents and young adults

**DOI:** 10.1007/s10147-022-02154-4

**Published:** 2022-03-26

**Authors:** Norio Yamamoto, Yoshihiro Araki, Hiroyuki Tsuchiya

**Affiliations:** Department of Orthopaedic Surgery, Graduate School of Medical Sciences, Kanazawa University, 13-1, Takaramachi, Kanazawa-city, Ishikawa 920-8641 Japan

**Keywords:** Adolescents and young adults, Bone sarcoma, Joint preservation surgery

## Abstract

Bone sarcoma often occurs in childhood, as well as in adolescents and young adults (AYAs). AYAs differ from pediatric patients in that their bone is skeletally mature and the physis has almost disappeared with the completion of growth. Although AYAs spend less time outside, they often participate in sports activities, as well as driving, working, and raising a family, which are natural activities in daily living. Multidisciplinary approaches involving imaging, multi-agent chemotherapy, surgical procedures, and careful postoperative care has facilitated an increase in limb-sparing surgery for bone sarcoma. In addition, recent advances in imaging modalities and surgical techniques enables joint-preservation surgery, preserving the adjacent epiphysis, for selected patients following the careful assessment of the tumor margins and precise tumor excision. An advantage of this type of surgery is that it retains the native function of the adjacent joint, which differs from joint-prosthesis replacement, and provides excellent limb function. Various reconstruction procedures are available for joint-preserving surgery, including allograft, vascularized fibula graft, distraction osteogenesis, and tumor-devitalized autografts. However, procedure-related complications may occur, including non-union, infection, fracture, and implant failure, and surgeons should fully understand the advantages and disadvantages of these procedures. The longevity of the normal limb function for natural activities and the curative treatment without debilitation from late toxicities should be considered as a treatment goal for AYA patients. This review discusses the concept of joint-preservation surgery, types of reconstruction procedures associated with joint-preservation surgery, and current treatment outcomes.

## Introduction

Bone sarcoma often occurs in childhood, as well as in adolescents and young adults (AYAs) [1]. The bone in pediatric patients is skeletally immature with an open physis in the epiphyseal area, and surgical treatment may cause various problems associated with future growth [2, 3]. In contrast, the bone in AYA patients is skeletally mature and the physis has nearly disappeared with the completion of growth, and these patients often participate in sports activities, as well as driving, working, and raising a family, which are natural activities in daily living [4].

Advances in chemotherapy and multidisciplinary treatment have extended the lifespan of malignant bone tumor patients [5, 6]; thus, AYA patients are expected to be longer survivors than older adult patients. Consequently, long durability and a normal function of the affected skeleton is required.

**Fig. 1:**
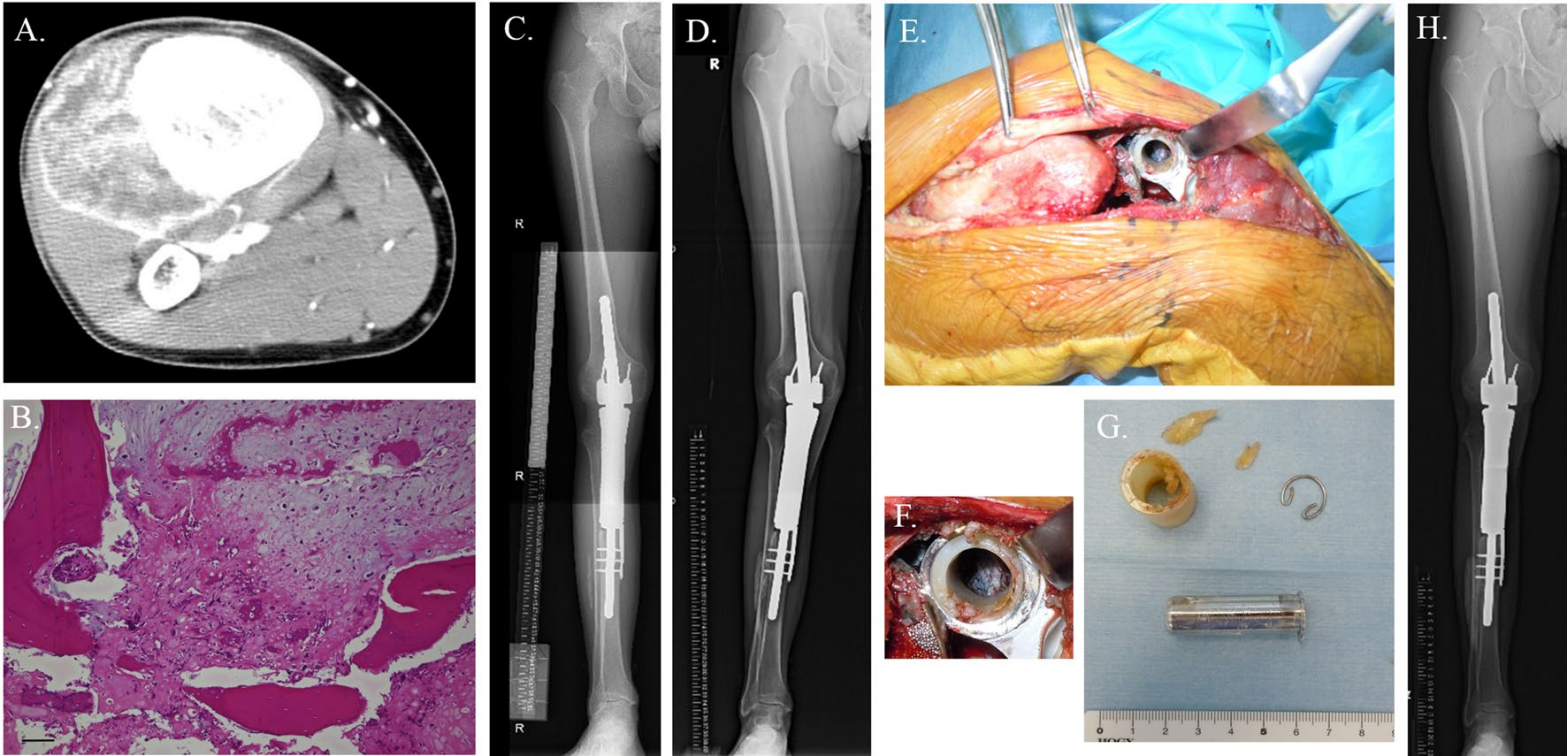
Artificial materials breakage for long-term period after primary surgery. A 16-year boy noticed his right knee pain. A screlotic bone tumor with extraosseous lesion was observed in the axial images of CT (**A**). Histology in a biopsy specimen was diagnosed with chondroblastic osteosarcoma of the right proximal tibia (a black bar shows 100 µm) (**B**). The patient underwent tumor prosthesis reconstruction with wide excision of the tumor after neoadjuvant chemotherapy. A neutral alignment of lower extremity was retained on standing X-ray at 8 years after primary surgery (**C**). At 15 years postoperatively, the valgus deformity and instability of the knee joint appeared due to an artificial material breakage (**D**). A bush component breakage with polyethylene wear and metallosis was observed in the revision surgery (**E**, **F**, **G**). The involved broken components were newly exchanged. He is alive with no evidence of disease at 24 years after the primary surgery, and work as a pharmacist (**H**)

In the surgical treatment of bone sarcoma, limb salvage surgery has replaced limb amputation [7]. Limb salvage surgery included various procedures; however, tumor prosthesis replacement after tumor excision is still mainstream [8]. Bone defect augmentation with artificial materials may produce good functional short-term recovery. Over time, the durability of artificial materials becomes an issue; thus, artificial materials cannot be considered a permanent solution for reconstruction (Fig. 1).

Regaining the function, permanence, and form as close to normal as possible are the long-term goals. Thus, the use of physiological materials is desirable. Joint-preservation surgery for biological reconstruction is, therefore, essential for retaining a normal limb function of the affected joint and satisfaction of highly active AYA patients.

Joint-preserving surgery for bone sarcoma involves various procedures. This review introduces biological procedures for joint preservation, including novel three-dimensional (3D) printing techniques.

## Joint-preserving surgery

Joint-preserving surgery allows patients to retain the original joint function, and is performed for selected patients. Candidates are selected among patients who opt for joint salvage surgery when the lesion has a favorable response to chemotherapy without pathologic fracture or extrusion of the tumor into the joint, and no whole-epiphyseal osteolytic area, large extraosseous mass, or obvious neurovascular involvement [9].

Takeuchi et al. reported that joint-preservation surgery involves three types of excision based on preoperative MRI; transmetaphyseal excision, transphyseal excision, and transepiphyseal excision. Among these, transepiphyseal excision is the most complicated and accurate osteotomy techniques are required [10]. Bosma et al. compared the accuracy of bone resection in knee joint-preservation surgery in four fresh-frozen human cadavers between freehand and intraoperative guidance technique groups, including patient-specific instrumentation (PSI) and computer-assisted surgery (CAS). They concluded that PSI showed the best resection accuracy (1.9 ± 1.1 mm) and provided the fastest cutting time (4.8 ± 1.0 min), in comparison to the freehand group, CAS group, and CAS + PSI group [11]. Wong et al. reported that joint-preserving tumor surgery under image-guided computer navigation in 8 patients (mean age, 17 years; mean follow-up period, 41 months) and found that accurate resection was achieved with ≦2-mm difference in any dimension, and with no local recurrence. The mean Musculoskeletal Tumor Society (MSTS) score was 29 points [12]. Abe et al. compared the satisfaction and function between joint-replacement and joint-preservation groups, and reported better physical outcomes and higher satisfaction in the joint-preservation group, based on the MSTS score and Toronto Extremity Salvage Score (TESS) [13, 14].

Several biological reconstruction methods for joint-preservation surgery have been developed, including allografts, vascular fibular grafts, distraction osteogenesis, and tumor-devitalized autografts. Table 1 summarizes the advantages and disadvantages of various joint-preserving surgery reconstruction methods, including allograft, vascularized fibula graft, distraction osteogenesis, and tumor-devitalized autografts.

**Table 1:** Advantages and disadvantages of various joint-preservation surgery reconstruction methods

	Allograft	Vascularized fibula	Distraction osteogenesis	Tumor-devitalized autografts
Advantages	Reattachment of a capsule, ligament and tendon	Short operative time Easy operative technique Remodeling capacity at the donor site Retaining of the ability of living bone (e.g., blood supply)	Gradual reconstruction with regenerative bone formation Longevity and durability	Perfect fit to the original site No risk of viral transmission or immune response problems Easy attachment of capsules, ligaments and tendons Preservation of bone stock Biological stability after graft union Absence of donor-site morbidity
Disadvantages	High rates of complications (e.g., infection, fracture, non-union) Impaired growth	High risk of complications (e.g., fracture, nonunion, and infection)	Cosmetic problems associated with external fixator Some complications (e.g., infection, joint contracture, and nonunion)	Some complications (e.g., infection, fracture, non-union, and bone absorption)

## Allograft reconstruction

**Fig. 2:**
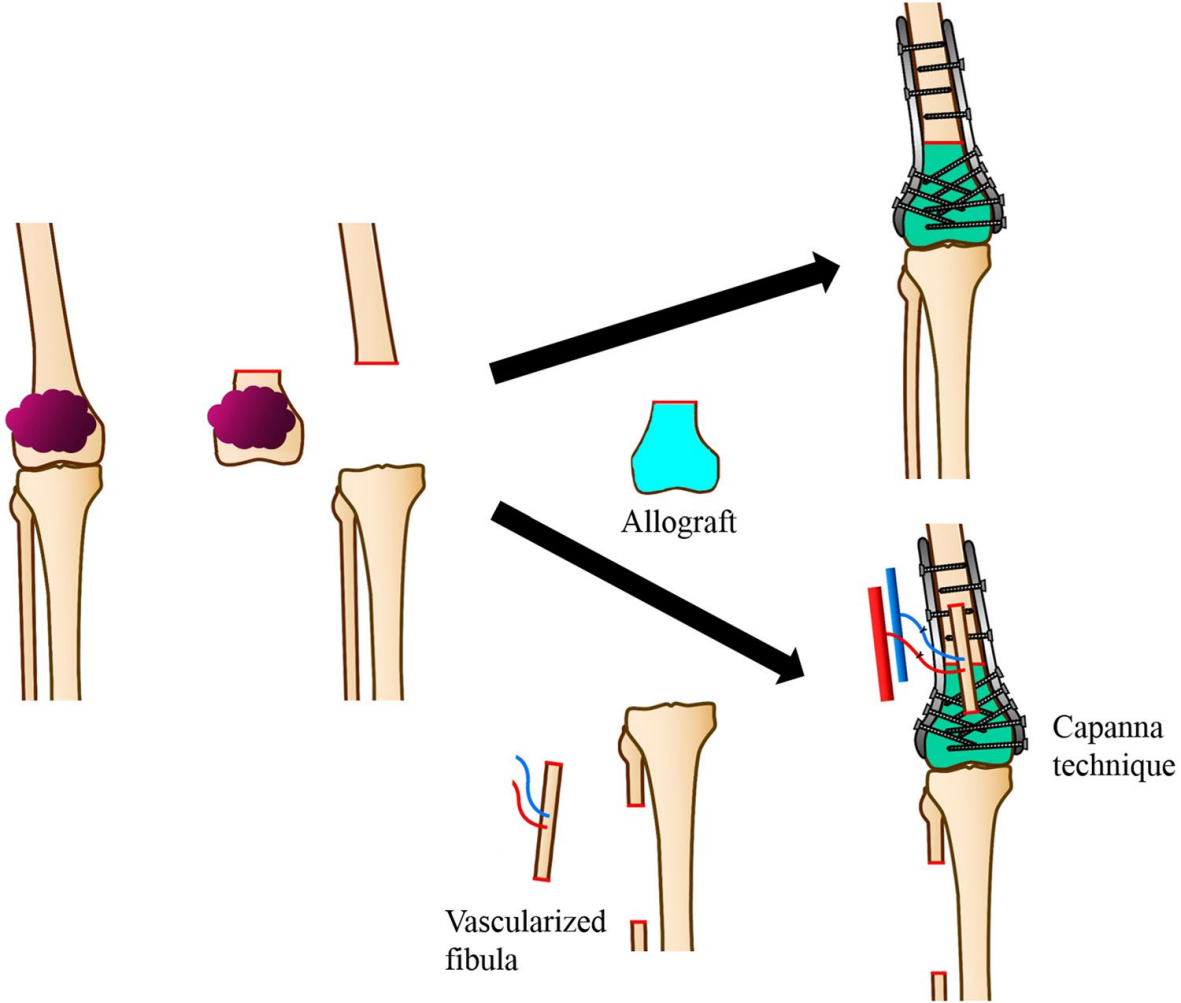
Allograft reconstruction method. After wide excision of the tumor was performed, bone defect is reconstructed using an allograft obtained from a bone bank or ultra-deep temperature freezer. The allograft and host bone are fixed with double plates, and sometimes combining with vascularized fibula graft (Capanna technique)

Bauer et al. first described the transplantation of bones stored by refrigeration in 1910, and fresh frozen allografts have been used for bone defect augmentation after tumor excision [15–17]. Allografting enables reattachment of a capsule, ligament and tendon to the graft, and incorporation of the host-allograft junction can be expected [18]. Disadvantages include high rates of infection, fracture, non-union, and impaired growth [19, 20]. This reconstructive method is described in Fig. 2.

Aponte-Tinao et al. reported the long-term clinical outcomes of 198 patients (mean follow-up period, 222 months), noting that 56% of transplanted allografts were retained for > 10 years. The risk of removal increased with time, even at 10 years, and 58% of the osteoarticular tibial allograft was commonly removed at 20 years. Infection of the tibia and fracture of the femur frequently occurred [21]. Albergo et al. investigated 71 patients (mean age, 16 years) who underwent intercalary femur reconstruction using allografts. The failure probability was 22% at 5 years, and fracture and nonunion occurred in 17 and 4 patients, respectively. The mean MSTS score was 27 points [22]. Errani et al. described 81 patients (mean age, 13.4 years) who underwent reconstruction using intercalary allograft and vascularized fibula. With a mean follow-up period of 96 months, the graft survival rate was 94%, and good to excellent function (according to the MSTS score) was achieved in 91% of cases. Fracture and infection were observed in 19 and 5 patients [23]. Campanacci et al. described reconstruction with massive allograft and a vascularized fibular graft in 23 patients (mean age, 16 years), reporting graft survival rate of 94.4% at 15 years and MSTS score of 94%. Fracture and nonunion were observed in 5 and 3 patients, respectively [24]. Othman et al. reviewed 25 articles on the utilization of an allograft alone (12 articles) and an allograft with an intramedullary vascularized fibula graft (13 articles). The allograft and vascularized fibula graft group had significantly lower rates of nonunion (13% vs. 21.4%). The infection (7.9% vs. 9%) and fracture (19.6% vs. 19.1%) rates were similar. The explantation rate was significantly higher in the allograft alone (18.1% vs. 6.6%) [25]. Table 2 summarizes the clinical outcomes of allograft reconstruction in previous studies, including graft survival, complications, and the postoperative function.

**Table 2:** Allograft reconstruction method and the clinical outcomes in previous studies

Study, year	Reconstruction	No. of patients	Mean age (range)	No. of AYAs	Location	Histology	Mean follow-up period (range)	Graft survival /years	Complications	MSTS score
Aponte-Tinao LA, 2020 [21]	Allograft	198	22 (2–55)	n.a.	Femur(132), Tibia(66)	OS(125), ES(19), CS(10)	222 months (120–370)	60%/10 years, 56%/20 years	Fracture(15%), Infection(14%), Nonunion(12%)	n.a.
Albergo JI, 2020 [22]	Allograft	71	16 (6–55)	n.a.	Femur(71)	OS(44), ES(16), CS(8)	129 months (12–311)	EFS 61%/10 years	Fracture(17), Nonunion(4), Infection(1)	93%
Errani C, 2019 [23]	Allograft + vascularized fibula graft (Capanna technique)	81	13.4 (n.a.)	n.a.	Femur(33), Tibia(48)	OS(46), ES(31)	96 months (26–265)	94%/(n.a.)	Fracture(19), Infection(5)	Good to excellent 91%
Campanacci DA, 2018 [24]	Allograft + vascularized fibula graft (Capanna technique)	23	16 (5–40)	4	Femur(23)	OS(11), ES(8), CS(2)	141 months (24–313)	94.4%/15 years	Fracture21.3%, Nonunion13%	94%

*AYAs* Adolescents and young adults, *No.* Numero (Number), *MSTS* Musculoskeletal Tumor Society

## Non-vascularized/vascularized fibula graft reconstruction

Non-vascularized fibula grafts have been used for biological reconstruction after the excision of a musculoskeletal tumor since the beginning of the twentieth century [26]. Advantages of this procedure consist of a short operative time, an easy operative technique, and a remodeling capacity at the donor site; however, disadvantages include high risk of absorption, fracture, nonunion, and infection [27, 28].

**Fig. 3:**
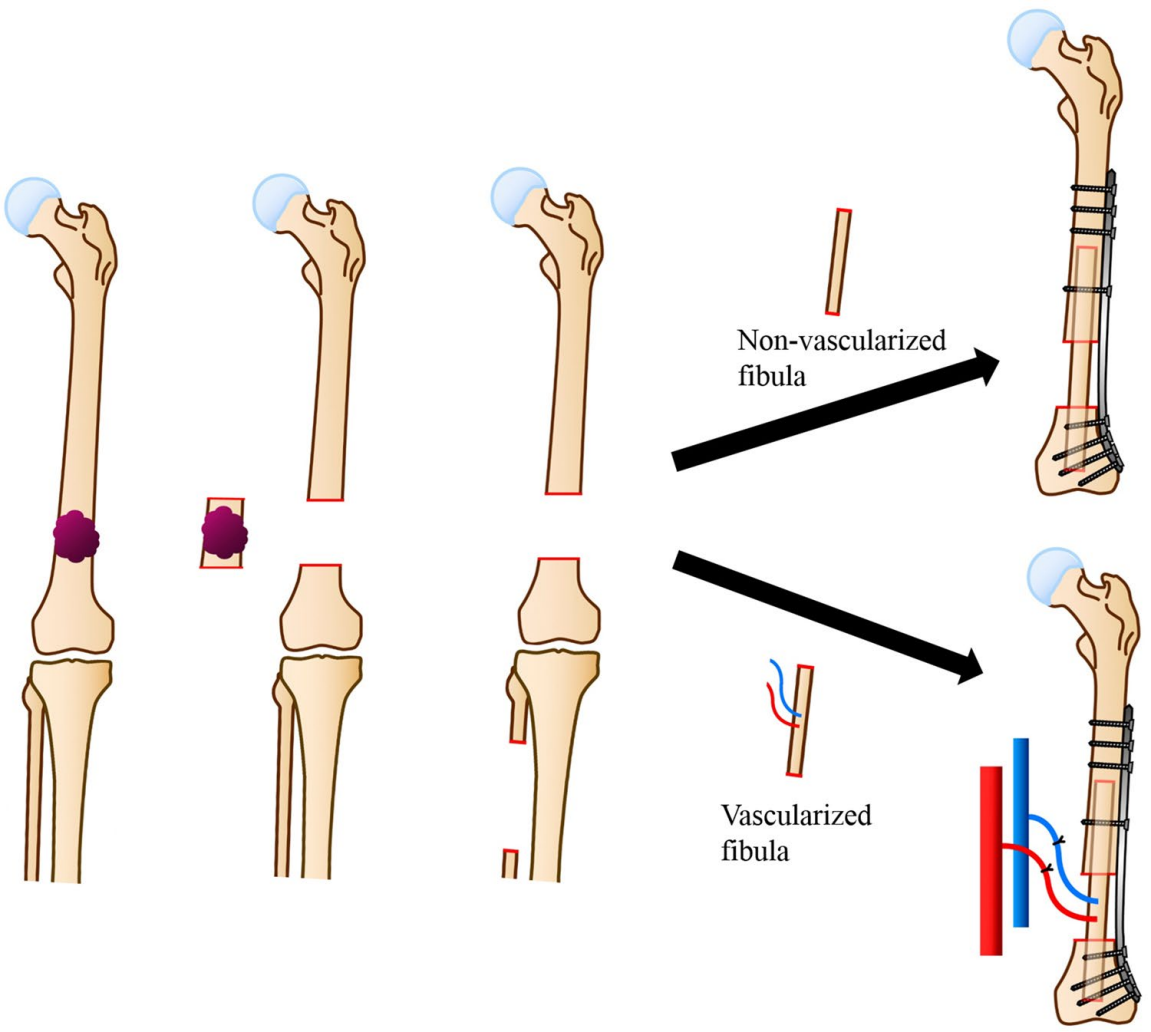
Non-vascularized/Vascularized fibula graft reconstruction method. After wide excision of the tumor was performed, bone defect is reconstructed using non-vascularized fibula graft or vascularized fibula graft, which is fixed with single or dual plates

On the other hand, vascularized fibula grafts can retain the ability of living bone (e.g., blood supply), and thus, bone resorption was absent. Taylor et al. first described this technique for trauma in 1975, and Weiland et al. applied it for reconstruction following tumor excision in 1977 [29, 30]. The disadvantages include non-union, malunion, stress fracture, infection, and donor-site comorbidity. This reconstructive method is described in Fig. 3.

Kreig et al. reported that, after pelvic reconstruction with non-vascularized fibula graft, primary union was achieved with a mean time of 27 weeks in 16 of 18 patients (mean age, 37.3 years; mean follow-up, 10.14 years). Nonunion, fracture, and wound infection occurred in 3, 5, and 2 patients, respectively [31]. Schuh et al. compared 26 patients with a vascularized fibular graft and 27 patients with a non-vascularized fibular graft for the reconstruction of a diaphyseal bone defect. Nonunion was observed in 3 with vascularized fibula graft and in 9 with non-vascularized fibula graft, while wound breakdown was observed in 10 with vascularized fibula graft and in 2 with non-vascularized fibula graft [32].

Houdek et al. reviewed 109 patients (mean age, 33 years) who received a free vascularized fibula graft to treat large bony defects (mean length 16 cm; range 6–30 cm). The bone union rate at 2 and 5 years was 82% and 97%, respectively. Tobacco use significantly increased the risk of nonunion in comparison to a locking plate, supplementary allograft, or > 16 cm graft [33]. Houdek et al. reviewed 24 cases (mean age, 37 years) who underwent reconstruction for segmental defects of the spine and pelvis, using a free fibular graft. The bone union rate was 86% at a mean time of 7 months. Common complications included wound dehiscence (*n* = 6), deep infection (*n* = 5), hardware failure (*n* = 4), and graft fracture (*n* = 3) [34]. Landau et al. reviewed 365 patients (average age 24.9 years) in 56 studies on vascularized fibula grafting after upper extremity tumor resection. The bone union rate was 93.3% with a median time of 5 months. Common complications included fracture (11.7%), nerve palsy (7.5%), infection (5.7%), and hammer toe deformity (3.3%). The median MSTS score was 80% [35]. Table 3 summarizes the clinical outcomes of non-vascularized/vascularized fibula graft reconstruction in previous studies, including graft survival, complications, and the postoperative function.

**Table 3:** Non-vascularized/vascularized fibula graft reconstruction method and the clinical outcomes in previous studies

Study, year	Reconstruction	No. of patients	Mean age (range)	No. of AYAs	Location	Histology	Mean follow-up period (range)	Graft survival /years	Complications	MSTS score
Lenze U, 2017 [27]	Non-vascularized fibula	36	24 (6–68)	22	Femur(20), Tibia(5), Humerus(6), Forearm(3), Fibula(2)	OS(2), ES(2), CS(9)	8.3 years (2.1–26.6)	n.a.	Fracture(5), Infection(2)	86%
Schuh R, 2014 [32]	Non-vascularized fibula	27	22.5 (3.6–62.4)	n.a.	Femur(1), Tibia(19), Humerus(2), Forearm(4)	OS(7), ES(7)	67.2 months (12–258.8)	n.a.	Nonunion(9), Fracture(2), Wound breakdown(2)	76%
Landau MJ, 2018 [35]	Vascularized fibula	365	24.9 (n.a.)	n.a.	Humerus(57.3%), Radius(36.2%), Ulna(5.8%)	OS(35.1%), CS(17.7%), ES(11.1%)	57.7 months (14–210)	n.a.	Fracture 11.7%, Nerve palsy 7.5%, Infection 5.7%, Hammer toe deformity 3.3%	80%
Houdek MT, 2017 [34]	Vascularized fibula	24	37 (13–68)	10	Spine(7), Spinopelvicregion(13), Pelvis(4)	OS(7), CS(5), ES(3)	5 years (1–15)	55%/5 years, 37%/10 years	Wound dehiscence(6), Deep infection(5), Hardware failure(4), Fracture(3)	53%

*AYAs* Adolescents and young adults, *No.* Numero (Number), *MSTS* Musculoskeletal Tumor Society

## Distraction osteogenesis

**Fig. 4:**
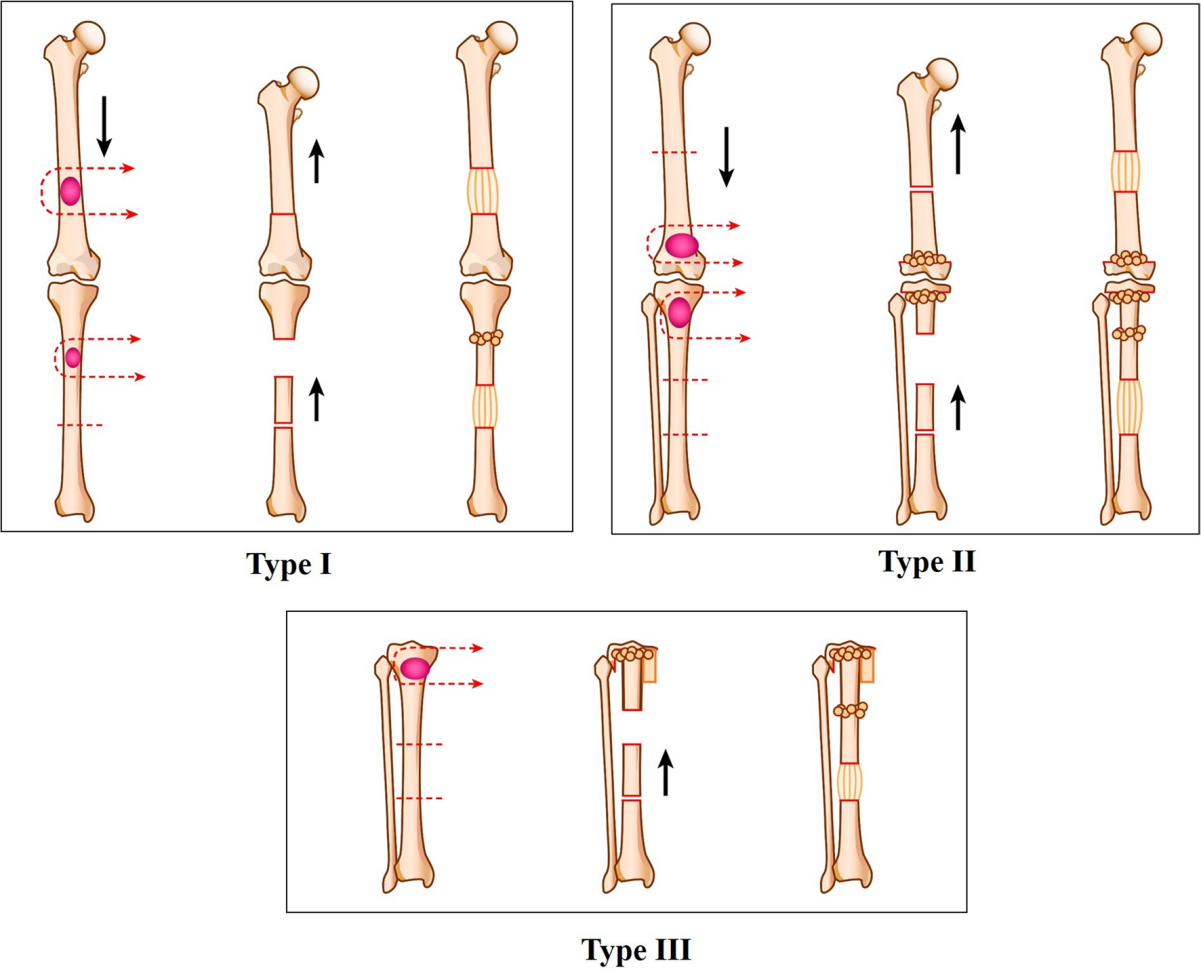
Classification of joint-preservation surgery using distraction osteogenesis. Type I: diaphyseal reconstruction. Shortening-distraction procedure was applied in case of femur when the tumor at the diaphyseal site was excised with an adequate margin. Bone transport procedure using bone cylinder from the proximal side of the remaining bone, was applied in case of tibia when the tumor at the diaphyseal site was excised with an adequate margin. Type II: metaphyseal reconstruction. Distraction procedure using bone cylinder from the proximal side of the diaphyseal bone by one osteotomy was applied in case of femur when the tumor at the metaphyseal site was excised with a clear margin and filled the metaphyseal bone defect with shortening the remaining bone and an iliac bone graft. Bone transport procedure using bone cylinder from the distal side of the diaphyseal bone cut by two osteotomy was applied in case of tibia after the wide excision of the tumor at the metaphyseal site was performed and filled the metaphyseal bone defect with shortening using bone cylinder from the proximal side of the diaphyseal bone cut by two osteotomy and an iliac bone graft. Type III: subarticular reconstruction. Bone transport procedure using bone cylinder from the distal side of the diaphyseal bone cut by two osteotomy was applied in case of tibia after the subarticular excision of the tumor was performed and filled the subarticular bone defect with shortening using bone cylinder from the proximal side of the diaphyseal bone cut by two osteotomy and an iliac bone graft

The standard indications for a bone transport procedure are a metaphyseal or diaphyseal defect with a preserved joint surface. Distraction osteogenesis is a biological method utilized for gradual reconstruction with regenerative bone formation using an external fixator. Canadell et al. first described the idea of physeal distraction for juxtaphyseal tumors [36]. Tsuchiya et al. classified five types of reconstructive strategies using external fixation based on tumor location. Among them, joint-preservation surgery included diaphyseal reconstruction (type I), metaphyseal reconstruction (type II), and subarticular reconstruction (type III) (Fig. 4) [37].

Tsuchiya et al. also described 11 cases (mean age 21.5 years) treated by distraction osteogenesis after knee joint-preserving excision of osteosarcoma. The mean MSTS score was 97.8% and 10 cases achieved full-range knee joint movement [38]. Watanabe et al. analyzed 22 patients (mean age 25.3 years) with a mean follow-up period of 202 months. The average MSTS score was 91.5%, and 14 patients could play sports without any difficulty [39]. McCoy et al. studied 20 patients (mean age 22.6 years) who underwent distraction osteogenesis with a mean follow-up period of 81.7 months. The mean MSTS scores for the lower and upper extremities were 93% and 87%, respectively [40]. Demiralp et al. described 13 patients (mean age 19.5 years; mean follow-up period 157 months), and noted that the mean MSTS score was 89.5%. Infection, joint contracture, and nonunion were observed in 8, 5, and 3 cases, respectively [41]. Wang et al. reported that 10 patients (mean age 14 years; mean follow-up period, 68.6 months) showed a mean MSTS score of 90%. Malunion (*n* = 3), malalignment (*n* = 2), and infection (*n* = 2) were observed [42]. Lesensky et al. reported that reconstruction with distraction osteogenesis involves an initial decreased function and longer recovery in comparison to other reconstructive techniques. This shortcoming is potentially outweighed by the longevity, function and durability of this method [43]. Moreover, an intramedullary lengthening nail can be advantageous for diaphyseal defects, because it maintains the alignment of the transporting bone segment, eliminates soft tissue scarring from pins or wires and the risk of pin tract infection; thus, yielding better cosmetic results. Accadbled et al. reported that a motorized intramedullary nail in 8 patients (mean age 11 years; mean follow-up period 30.5 months) represents a safe and reliable procedure for bone defects with a mean length of 15.5 cm. The mean MSTS score was 79.9% and loosening of a locking screw was observed in 2 cases [44]. Table 4 summarizes the clinical outcomes of the distraction osteogenesis reconstruction method in previous studies, including graft survival, complications, and the postoperative function.

**Table 4:** Distraction osteogenesis reconstruction method and the clinical outcomes in previous studies

Study, year	Reconstruction	No. of patients	Mean age (range)	No. of AYAs	Location	Histology	Mean follow-up period (range)	Graft survival /years	Complications	MSTS score
Wang W, 2019 [42]	Distraction osteogenesis	10	14 (11–51)	4	Femur (7), Tibia (3)	OS(10)	68.6 ± 26.6 months	90%/(n.a.)	Malunion(3), Malalignment(2), Infection(2)	90%
Demiralp B, 2014 [41]	Distraction osteogenesis	13	19.46 (7–42)	8	Femur (5), Tibia (8)	OS(7) ES(3)	157.23 months	100%/(n.a.)	Infection(8), Contructure(5), Nonunion(3)	89.46%
Watanabe K, 2013 [39]	Distraction osteogenesis	22	25.3 (8–54)	10	Femur (7), Tibia (15)	OS(7) ES(1)	202 months	100%/(n.a.)	Delayed union(4), Contracture(3), Fracture(2), Infection(2)	91.5%

*AYAs* Adolescents and young adults, *No.* Numero (Number), *MSTS* Musculoskeletal Tumor Society

## Tumor-devitalized autograft reconstruction

Tumor-devitalized autograft reconstruction is a unique biological procedure involving reimplantation of tumor-bearing bone after the following devitalized treatment: extracorporeal irradiation [45–51], pasteurization [52–57], and freezing [58–66]. The advantages of the autograft include a perfect fit to the original site, no risk of viral transmission or immune response problems, easy attachment of capsules, ligaments and tendons, preservation of bone stock, biological stability after graft union, and absence of donor-site morbidity. However, some complications (e.g., infection, fracture, non-union, and bone absorption) were reported. Table 5 summarizes the clinical outcomes of the various tumor-devitalized autograft reconstruction methods in previous studies, including graft survival, complications, and the postoperative function.

**Table 5:** Various tumor-devitalized autograft reconstruction methods and the clinical outcomes in previous studies

Study, year	Reconstruction	No. of patients	Mean age (range)	No. of AYAs	Location	Histology	Mean follow-up period (range)	Graft survival /years	Complications	MSTS score
Outani H, 2020 [47]	Irradiated	56	20 (6–76)	n.a.	Femur (17), Tbia (22) humerus (13)	OS(32), ES(5) CS(5)	16.5 years (10.1–26.2)	82.1%/10 years 76.8%/15 years	Structural failure(12), Skin irritation(8), Infection(7), Fracture(3)	80%
Oike N, 2019 [48]	Irradiated	27	31.7 (9–59)	6	Femur (13), Tibia (7)	OS(10) CS(6)	16.6 years (10.3–24.3)	88.9%/10 years	Nonunion(9), Subchondral bone collapse(4), Deep infection(4)	84.3%
Wu PK, 2018 [49]	Irradiated	79	19 ± 10	n.a.	Femur (46), Tibia (14) Humerus (4)	OS(79)	82 ± 54 months	83%/5 years	Wound complication(6), Nonunion(8), Fracture(2), Implant failure(1), Infection(6)	n.a
Lee SY, 2018 [53]	Pasteurized	278	24 (5–72)	n.a.	Femur(137), Tibia(81) Humerus(34)	OS(201), ES(11), CS(23)	113 months (25–295)	59%/10 years 40%/20 years	Infecction13%, Nonunion7%, Fracture6%, Bone absorption6%	n.a
Ikuta K, 2018 [55]	Pasteurized	24	22 (5–61)	5	Femur(17), Tibia(1) Humerus(4)	OS(20)	88 months (28–208)	70.1%/5 years 70.1%/10 years	Nonunion(14), Infection(3), Bone absorption(5), fracture(1)	76%
Sugiura H, 2012 [54]	Pasteurized	46	30.7 (10–78)	30	Femur(15), Tibia(10), Humerus(2)	OS(17), ES(3), CS(5),	8.7 years (2–17)	94%/10 years	Nonunion17%, Fracture15%, Infection13%, Bone absorption13%	83.8%
Araki Y, 2021 [65]	Frozen	37	23 (15–39)	37	Femur(20), Tibia(11), Humerus(5), Calcaneus(1)	OS(29), ES(3), UPS(3)	89 months (5–229)	86%/5 years, 76%/10 years	Infection(7),OA(5), Bone absorption(3), Fracture(2), Nonunion(1), Deformity(1)	Excellent 79%
Wu PK, 2018 [49]	Frozen	85	20 ± 12	n.a.	Femur(38), Tibia(24) Humerus(5)	OS(85)	70 ± 25 months	84%/5 years	Wound complication(3), Nonunion(11), Fracture(5), Implant failure(2), Infection(4)	n.a
Igarashi K, 2014 [61]	Froezn	36	39 (10–72)	13	Femur(16), Tibia(9), Humerus(3)	OS(19), CS(5)	101 months (61–163)	80.6%/10 years	Fracture(7), Infection(4), Nonunion(4)	Excellent 72.2%

*AYAs* Adolescents and young adults, *No.* Numero (Number), *MSTS* Musculoskeletal Tumor Society

### Extracorporeal irradiated autograft

Spira et al. first described extracorporeal irradiated bone transplantation in 1968. The excised tumor-bearing bone was extracorporeally irradiated (50–300 Gy) and reimplanted at the original position [45, 46].

Outani et al. reported that the 10-year graft survival rate was 82.1% in 56 patients (mean age 20 years; mean follow-up period 16.5 years). Structural failure, skin irritation, infection, and fracture were observed in 12, 8, 7, and 3 cases, respectively. The mean MSTS score was 80% [47]. Oike et al. reported that the 10-year graft survival rate was 88.9% in 27 patients (mean age 31.7 years; mean follow-up 16.6 years). Nonunion and infection occurred in 9 and 4 patients, respectively. The mean MSTS score was 84.3% [48]. Wu et al. described 79 patients (mean age 19 years), and bone union was achieved 87% at 18 months, nonunion was observed in 8 cases. Infection and recurrence were observed in 6 and 12 cases, respectively [49]. Jones et al. reviewed 113 patients (mean follow-up periods 80.3 months), noting that 92.9% of grafts remained in place, with failure due to complications in 8 patients. The primary union rate was 65% at a mean time of 15 months, and nonunion and delayed union were observed in 6% and 17% of cases, respectively. Infection and recurrence were observed in 9% and 9% of cases, respectively. The mean MSTS score was 79% [50]. Mihara et al. described 15 patients (mean age 33.7 years; mean follow-up period 71.8 months) who received an extracorporeally irradiated autograft and vascularized fibula graft, reporting a graft survival rate of 93.3%. Bone union was achieved in 12 of 14 patients (mean period 10.8 months). Nonunion and infection were observed in 2 and 2 patients, respectively. No fracture was observed due to high mechanical strength. The mean MSTS score was 82.7% [51].

### Pasteurized autograft

In pasteurized tumor-devitalized autograft reconstruction, tumor-bearing bone is heat-sterilized at 60–65 °C for 30–40 min. Manabe et al. began developing this procedure in 1990, and reported the first clinical outcomes of 25 patients (mean age 24 years; mean follow-up period 52 months) in 2004. Complete incorporation was obtained in 60%. The mean MSTS score was 86%. Infection and fracture were observed in 20% and 12% of patients, respectively [52].

Lee et al. reported that 10- and 20-year graft survival rates were 59% and 40%, respectively, in 278 patients (average age 24 years; mean follow-up period 113 months). The survival in the patients who underwent osteoarticular or hemicortical graft reconstruction was better than that of patients who underwent prosthesis composite, intercalary, or fusion types of reconstruction. The rate of union at < 2 years was 56%, and the nonunion rate was 7%. Infection, fracture, and recurrence were observed in 13%, 6%, and 4% of patients, respectively [53]. Sugiura et al. reported that the 10-year graft survival rate was 94% in 46 patients (average age 30.7 years; mean follow-up period 8.7 years). The average time to bone union was 9.5 months. Nonunion, fracture, infection, and bone absorption, occurred in 17%, 15%, 13%, and 13% of patients, respectively. The average MSTS score was 83.8% [54]. Ikuta et al. reviewed 24 patients who underwent reconstruction for segmental bone defects in an extremity. Nonunion was observed in 18 of 48 junctions of autografts. Upper extremity defects showed significant associations with nonunion and bone absorption, and intercalary autograft without vascularized fibula was significantly associated with bone absorption. The graft survival rate was 70.1% at 10 years [55]. Nishida et al. histologically assessed the reparative process of pasteurized bone from 10 retrieved specimens by calculating the ratio of the number of viable cells to whole cells. Markedly better repair was promoted in pasteurized bone combined with a vascularized fibula in comparison to pasteurized bone without a vascularized fibula [56]. Liu et al. evaluated the efficacy of reconstruction with pasteurized autografts and a vascularized fibula grafts for femoral diaphyseal bone defects in 15 patients (mean age 22.3 years; mean follow-up period 65.1 months). Bone union was achieved in all patients. The mean time to union at the proximal and distal junctions was 8.7 and 9.2 months, respectively, for vascularized fibula grafts, 14.3 and 15.6 months, respectively, for pasteurized bone. Fracture of pasteurized bone was observed in 2 patients. No infection or local recurrence was observed. The mean MSTS score was 81.8% [57].

### Frozen autograft

**Fig. 5:**
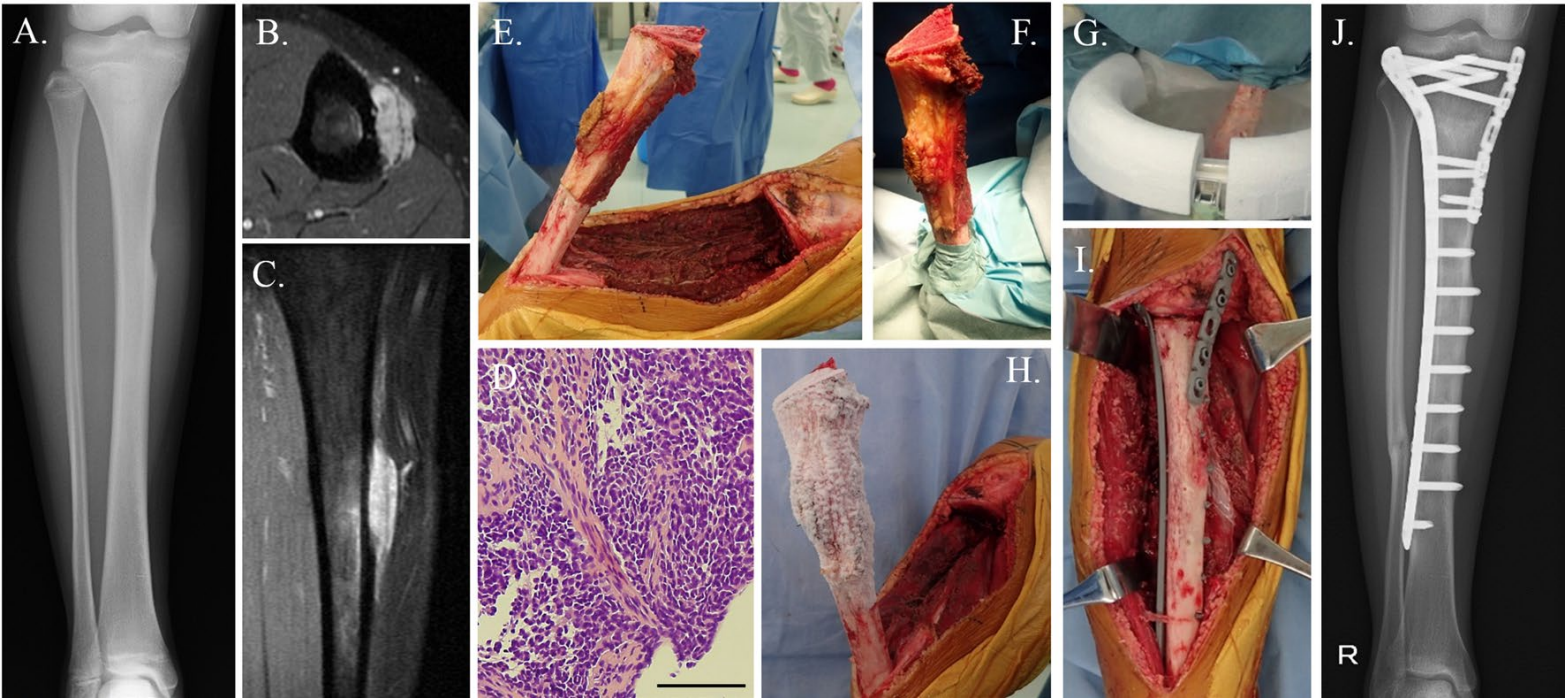
Frozen autograft reconstruction (Pedicle freezing procedure). A 15-year-old female patient noticed the swelling of the right tibia. A moth-eaton appearance in the right tibia shaft was observed on X-ray (**A**). The extraskeletal lesion with the infiltration of the cortical bone and the intramedullary bony edema was observed on T2 fat-suppression MR axial (**B**) and coronal (**C**) images. The histology in a biopsy specimen revealed with Ewing sarcoma (**D**). Chemotherapy was performed and almost complete remission of the lesion was achieved, and thus, surgical treatment was planned. The lesion was excised with adequate margins and the excised tumor-bearing bone was carefully elevated (**E**). The healthy tissues were carefully protected using several surgical sheets for protection of the contamination of tumor tissues on healthy body (**F**). After removal of the surrounding soft tissues and curettage of the intramedullary tumor, the residual bone was turned upside down and frozen for 20 min in liquid nitrogen that was stored in sterilized flask (**G**), and then thawed at room temperature for 15 min (**H**), and in a solution of 0.3% iodine and distilled water for another 15 min. The frozen autograft was fixed to the residual healthy bone on the original site with double locking plates (**I**). At 5 years after surgery, the bone union was achieved, and the function of the knee joint was perfectly preserved (**J**)

Tsuchiya et al. developed this procedure of recycling frozen tumor-bearing bone treated with liquid nitrogen, and it has been applied in the clinical setting since 1999 [58]. The bone strength and cell-killing effect after the freezing treatment were confirmed in basic research [59]. There are two main procedures, free freezing and pedicle freezing, which differ from other devitalized procedures [60]. The bone tumor is excised *en* bloc (free freezing) or with a pedicle to the healthy bone (pedicle freezing, Fig. 5). The bone is frozen for 20 min in liquid nitrogen, then thawed at room temperature for 15 min, and in 0.35% iodine distilled water at 30 °C for another 15 min.

Igarashi et al. reported 36 patients (mean age 39 years; mean follow-up period 101 months), with a graft survival rate of 80.6%. The MSTS score was excellent in 26 cases, and fracture, infection, and nonunion, were observed in 7, 4, and 4 cases, respectively. The long-term outcome was satisfactory in intercalary reconstruction and autograft-composite reconstruction, while osteoarticular graft reconstruction failed in 7 of 16 cases because of fracture or infection [61]. Wu et al. reported 85 patients (mean age 20 years), and bone union was achieved in 79.8% at 18 months, while nonunion was observed in 11 cases. Infection and soft tissue recurrence were observed in 4 and 9 cases, respectively [49]. Yamamoto et al. reported that liquid nitrogen-treated bone was histologically well revitalized on every part of frozen bone retracted at 8 years after implantation [62]. Tanzawa et al. reported that the revitalized area of frozen bone histologically expanded with time in 6 extracted frozen bone specimens at 2–96 months after implantation [63]. Shimozaki et al. compared the clinical outcomes of the free freezing (*n* = 13) and pedicle freezing (*n* = 7) procedures, and reported that the mean time of bone union in the pedicle and free freezing procedures was 4.8 and 9.8 months, respectively. Fracture was observed in 2 patients who underwent the pedicle freezing procedure, while infection, joint destruction, and fracture were observed in 3, 2, and 1 patient who underwent the free freezing procedure [64]. Araki et al. investigated the clinical outcomes of 37 AYA patients, and reported that bone union was achieved earlier in comparison to older adult patients (8 months vs. 10 months), and that fracture (*n* = 2) and nonunion (*n* = 1) occurred less frequently in comparison to 27 pediatric patients (fracture, *n* = 8; nonunion, *n* = 3) [65]. Lu et al. reported that frozen autograft combined with a vascular fibular graft in 8 patients was associated with earlier bone union (mean period 8.4 months) in comparison to the Capanna technique and that no infection or nonunion occurred. The mean MSTS score was 90.3% [66].

## 3D Printing techniques

**Fig. 6:**
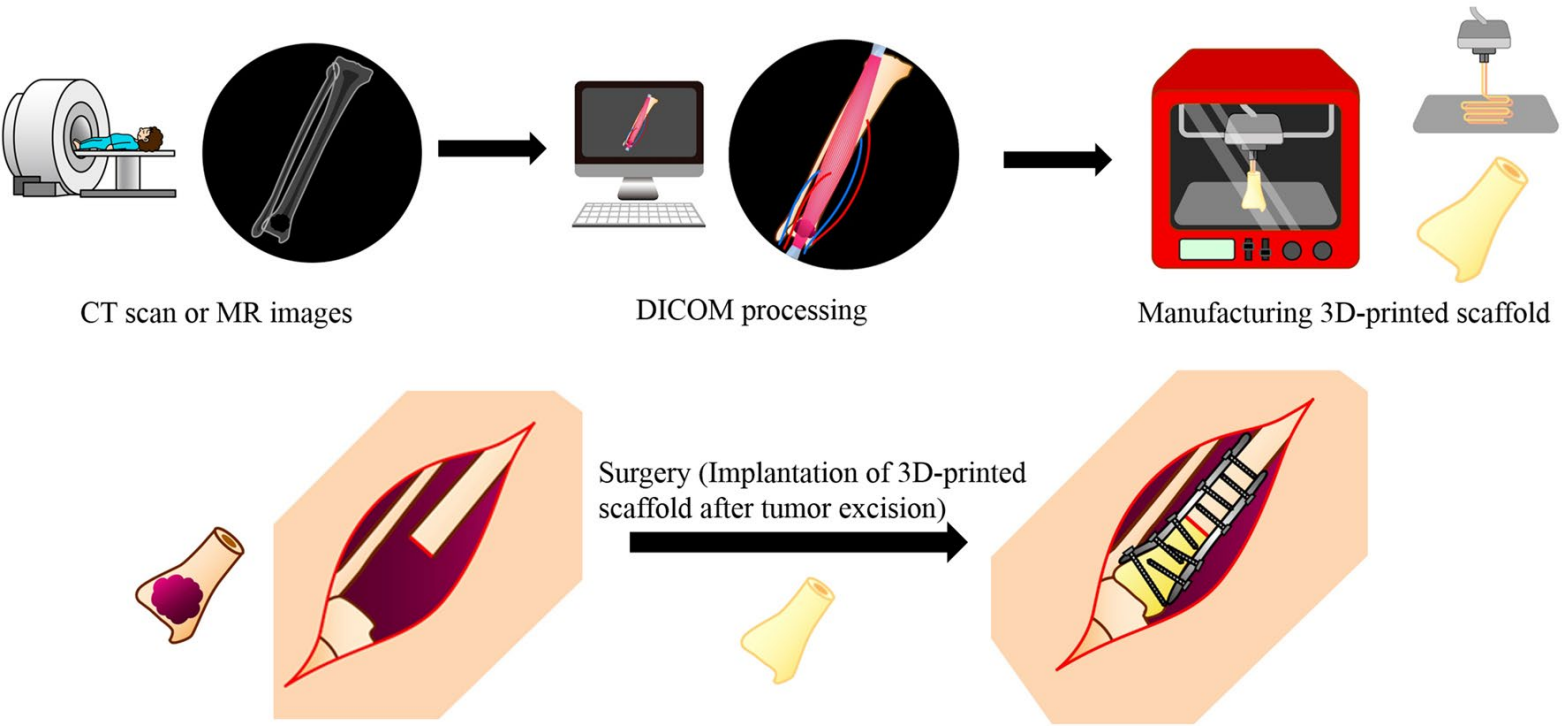
3D printing techniques. Based upon CT scan images or MR images of the patient, Digital imaging and communications in medicine (DICOM) processing was conducted using software to manufacture the 3D-printed scaffold. After wide excision of the tumor was performed, bone defect is reconstructed using the 3D-printed scaffold

3D printing technology, including bioprinting 3D medical models, has been developed since the 1990s [67]. This technology was applied in the clinical settings in orthopedic surgery and maxillofacial surgery, where it allows structures of bone defects to be reconstructed with 3D-printed patient-specific implants [68, 69]. The main advantage of 3D printing models is to allow the manufacture of customized scaffolds that mimic the patient’s precise anatomy, and printing accuracy was achieved by a robotic-assisted bioprinting techniques at an average dimensional error of 0.06 ± 0.14 mm [70]. Liu et al. described joint-preserving intercalary resection with the aid of 3D-printed osteotomy guide plates and reconstruction using 3D-printed intercalary prostheses in 12 patients with metaphyseal bone tumors around the knee joint. Accurate resection was achieved, and the mean MSTS score was 28 at an average follow-up period of 22.5 months [71]. Various types of ceramics (hydroxyapetite, beta- or alfa-tricalcium phosphate, biphasic calcium phosphate, bioactive glasses, etc.) have been used for 3D printing bone scaffolds. However, these bioceramics do not usually match mechanical strength for load-bearing. To obtain sufficient mechanical strength, bioceramics can be blended with polymers (cellulose or polycarprolactone); however, challenges remain for large-sized scaffolds [72, 73]. Moreover, bioprinting is a novel technique, wherein cell-laden hydrogels are used with the incorporation of angiogenic growing factors, endothelial cells, or tissue-specific cells into bio-inks, which can develop complex tissues, including bone or cartilage after maturation [74, 75]. Bioprinting will enable the production of customized and vascularized living bone; however, these biofabrication techniques are currently in the developmental stage, because there are still unresolved issues to overcome, specifically optimal cell numbers and viability, spatial 3D construction for cell differentiation, reconnection to the local vasculature, and cost. This reconstructive method is described in Fig. 6.

## Conclusions

This review discussed joint-preservation surgery, including various types of biological reconstruction. Reconstruction using megaprosthesis after bone tumor excision is still the standard treatment for all generations; however, prosthesis-associated complications increase with time, because the prosthesis has no self-repair mechanism. The longevity of the normal limb function for natural activities and curative treatment without debilitation due to late toxicities should be considered as a goal for the treatment of AYA patients. To realize this, we should select an optimal surgical procedure from the various joint-preservation procedures we reviewed, considering the psychosocial circumstances around the patient and his/her family, sports activities, driving, and working, in addition to tumor characteristics. We believe that sufficient attention must be given to the affected limb function and long-term complications, even after the completion of treatment of bone sarcoma in AYA patients.
